# Hepatitis B virus infection and HBeAg positivity among pregnant women in South West Uganda

**DOI:** 10.4102/ajlm.v11i1.1784

**Published:** 2022-08-18

**Authors:** Naome Mugabiirwe, Rogers Kalyetsi, Richard Ayella, James Obote, Frank Ssedyabane

**Affiliations:** 1 Department of Medical Laboratory, Kyazanga Health Centre IV, Lwengo, Uganda; 2 Department of Medical Laboratory Science, Faculty of Medicine, Mbarara University of Science and Technology, Mbarara, Uganda

**Keywords:** risk factors, hepatitis B, pregnant women, prevalence, Uganda

## Abstract

**Background:**

Hepatitis B virus is a public health burden in Uganda, yet little is known about its epidemiology in pregnancy.

**Objective:**

This study aimed at determining the prevalence and associated risk factors of hepatitis B virus infection among pregnant women attending antenatal care at the Kyazanga Health Centre IV in Lwengo District, Uganda.

**Methods:**

This cross-sectional study was conducted from April 2021 to June 2021 and analysed qualitative data that were collected using a structured in-person questionnaire. Aseptically collected blood specimens were screened for hepatitis B virus infection using an immunochromatographic rapid diagnostic test kit. Participants who were positive for the hepatitis B surface antigen (HBsAg) were further screened for hepatitis B envelope antigen (HBeAg) using commercial rapid diagnostic test kits.

**Results:**

Out of 384 pregnant women studied, eight tested positive for HBsAg. This gave a prevalence of 2.1% (95% confidence interval: 1.0% – 4.1%); 5/8 (62.5%) were positive for HBeAg. None of the variables studied were significantly associated with HBsAg positivity among pregnant women.

**Conclusion:**

Hepatitis B viral infection is still a public health challenge in pregnant women with possible risk for vertical transmission to their babies in the study area. We recommend routine screening for hepatitis B virus in pregnancy in addition to strengthening current strategies aimed at controlling and preventing hepatitis B infection spread and transmission.

## Introduction

Hepatitis B is caused by hepatitis B virus (HBV) infection. Hepatitis B virus belongs to the family Hepadnaviridae and is highly hepatotropic, causing acute and chronic liver diseases including cirrhosis and hepatocellular carcinoma.^1^ Hepatitis B virus is estimated to have caused 820 000 deaths worldwide^2^ by contributing to chronic hepatitis and hepatocellular carcinoma.^3^ The prevalence of HBV infection in sub-Saharan Africa is reported to range from 5% to 20%,^4^ with perinatal transmission estimate rates ranging from 1% to 5%.^4^

Uganda is known to be a highly endemic area of the infection with an estimated national prevalence of 10.0%.^5^ The regional distribution of disease varies, with the highest prevalence in the northern region; prevalence is 19% in the northwest and 25.0% in the northeast.^5^ The national prevalence of HBV infection among women attending antenatal care was 4.1% in the year 2018.^6^ Studies done in Uganda around 2018 estimated the prevalence of hepatitis B virus infection among pregnant women attending antenatal care to be 2.9% (95% confidence interval: 1.58% – 5.40%) at Mulago National Referral Hospital in central Uganda,^5^ 11.8% at two hospitals in northern Uganda^7^ and 3.1% at Mbarara Regional Referral Hospital in western Uganda.^6^ Some risk factors for hepatitis B infection include tattooing or use of contaminated sharp instruments, reuse of needles, intravenous drug use, percutaneous as well as mucosal exposure to infected blood or any other body fluids such as saliva, vaginal or seminal fluids.^5^

Mothers who are positive for hepatitis B surface antigen (HBsAg) have a 10% to 40% risk of passing the infection onto their newborn babies and the risk increases to 90% among mothers who are positive for the hepatitis B envelope antigen (HBeAg).^8^ Babies who are infected perinatally have a 90% risk of developing chronic infection.^9^ Antiviral therapy during late pregnancy and immunisation of babies exposed to HBV within the first 12 h of life may reduce mother-to-child transmission by 75% to 90% and reduce risk of developing chronic infection.^10,11^ The perinatal transmission risk is 70% to 90% for HBeAg-positive mothers, 25% for HBeAg-negative/anti-HBe-negative mothers and 12% for mothers who are positive for anti-HBe while negative for HBeAg.^5^

A number of studies have been conducted in south western Uganda on the epidemiology of HBV infection, though little data exists on prevalence and associated risk factors among pregnant women attending antenatal care at the Kyazanga Health Centre IV (HCIV). Therefore, this study aimed to determine the prevalence and risk factors associated with HBV infection among pregnant women attending antenatal care at Kyazanga HCIV, Lwengo District, South Western Uganda.

## Methods

### Ethical considerations

Ethical approval was sought from the Department of Medical Laboratory Science, Faculty Research Committee Faculty of Medicine Mbarara University of Science and Technology (MUST/MLS/030). Permission was also obtained from the District Health Officer, Lwengo District and the Office of Medical Officer Health Sub-District, Kyazanga Health Centre IV. Informed consent was obtained from all study participants. Participants were identified with numbers, not names, for prevention of breach of confidentiality. All completed questionnaires were archived in the Department of Medical Laboratory Science under lock and key. The generated computer database was kept inaccessible for non-study persons; for study personnel, access was restricted with a password.

### Study design and setting

This cross-sectional study was conducted between April 2021 and June 2021 at the Kyazanga HCIV Antenatal Clinic located in Kyazanga Town Council, Lwengo District in South Western Uganda. The Kyazanga HCIV is a public health facility which serves Buktoto west, including referrals from five other low-level health facilities.

### Sample size, study population and sampling strategy

We calculated a sample size of 384 participants based on a presumed 50% prevalence rate of HBV^6^ using an allowable standard error of ±0.05 at 95% level of confidence. The study population included pregnant women who attended the antenatal clinic during the study period. We used a systematic random sampling method to recruit all participants. Individuals were selected at regular intervals, where every second pregnant woman who arrived at the clinic was selected from the sampling frame.

### Data collection

With the help of clinic nurses, we administered an in-person structured questionnaire to collect socio-demographic data (age, place of residence, level of education, primary [completed primary school year seven], secondary [completed senior school year six], or tertiary [reached any tertiary institution]), participant’s place of birth and gravidity (primigravida [first pregnancy], multigravida [2–4 pregnancies], grand multigravida [≥ 5 pregnancies]) and risk factors for HBV (history of blood transfusion, history of abortion or miscarriage, history of hospital admission, history of tooth extraction, history of body piercing, history of unprotected sexual intercourse, history of sharing sharp materials and history of injection drug abuse) from study participants.

### Laboratory testing

Immediately after consent and completion of the questionnaire, three millilitres (3 mL) of venous blood was drawn aseptically by venipuncture from the mid-cubital vein, into ethylenediaminetetraacetic acid-vacutainers. Specimens were labelled using identification numbers (codes) and immediately taken to the laboratory for centrifugation (3000 rpm for 5 min) to separate plasma from blood cells. SD BIOLINE HBsAg Immunochromatographic rapid diagnostic test kits (Abbott Diagnostics Korea Inc, Gihueng-gu, Yongin-si, Republic of Korea) were used for qualitative detection of HBsAg in serum from each study participant.

Plasma was added to the sample pad on the HBsAg test strip and timed for 15 min for the reaction to occur. Results were then read, interpreted and recorded. Positive samples were then tested for the HBeAg, using the commercial SD BIOLINE HBeAg testing kit (Abbott Diagnostics Korea Inc, Gihueng-gu, Yongin-si, Republic of Korea) to show the level of infectivity and risk of infection. All laboratory testing was carried out within the health unit laboratory, following Standard Operating Procedures as well as manufacturer’s instructions.

### Quality assurance and control

The questionnaire was pre-tested and validated on five pregnant women attending antenatal care at the Kyazanga HCIV. Each batch of the rapid dipstick tests was pre-tested with positive and negative controls (known positive and known negative plasma samples) for quality assurance. Positive and negative controls were run along with each batch of samples. The sensitivity and specificity of the rapid test strip were 98.84% and 98.94%, respectively.

### Data management and analysis

Data were entered into a Microsoft Excel spread sheet (Microsoft Office Professional Plus 2013, version 15.0.4675.1003, Microsoft Inc, Redmond, Washington, United States) and then imported into STATA 13 (StataCorp LLC, College Station, Texas, United States) software for analysis. Demographic data were presented in the form of frequencies and percentages. Prevalence was presented as percentages and using pie charts. Associations between risk factors and hepatitis B were determined using regression analysis and a *p*-value of < 0.05 was considered to be statistically significant.

## Results

### Socio-demographic characteristics of pregnant women attending antenatal clinic at Kyazanga Health Centre IV

We recruited 384 pregnant women attending the antenatal clinic at Kyazanga HCIV from April 2021 to June 2021 (Table 1). The participants’ mean age was 27 years, with a standard deviation of 7.34 years. Participants’ ages ranged from 17 to 48 years, with the majority aged between 26 and 28 years. Most study participants were from urban areas (51.0%), had completed a primary seven level of education (65.0%), had been born in a hospital setting (61.2%) and were multigravidas (54.2%).

**TABLE 1:** Demographic characteristics of participants attending the antenatal clinic at Kyazanga Health Centre IV, South Western Uganda, between April 2021 and June 2021.

Variable	Category	Frequency (*n*)	Percentage
Age (year)	< 24	144	37.5
	24–35	181	47.1
	36–48	59	15.4
Place of residence	Rural	188	49.0
	Urban	196	51.0
Education level†	Primary	249	65.0
	Secondary	119	31.0
	Tertiary	16	4.0
Place of birth	Others	60	15.6
	Home	89	23.2
	Hospital	235	61.2
Gravidity‡	Primigravida	135	35.1
	Multigravida	208	54.2
	Grand multigravida	41	10.7

*n* = 384.

† , Primary, having finished primary grade seven; Secondary, having finished senior grade six; Tertiary, having reached any institution of higher learning.

‡ , Primigravida, first pregnancy; Multigravida, 2–4 full term pregnancies; Grand multigravida, ≥ 5 full term pregnancies.

### Prevalence of hepatitis B virus infection

Of the 384 participants, eight participants tested positive for HBsAg for an overall prevalence of viral hepatitis of 2.1% (Table 2). Of the eight HBsAg-positive participants, five tested positive for HBeAg (62.5% of HBsAg-positive participants). Of the eight participants who tested positive for HBsAg, 75.0% (6/8) were aged 24–35 years, 25.0% (2/8) were aged younger than 24 years (Table 3). Additionally, 62.5% (5/8) lived in an urban setting, and 37.5% (3/8) lived in a rural setting. A majority (62.5%; 5/8) had completed a primary seven level of education, 25.0% (2/8) had attained secondary education, 12.5% (1/8) had tertiary education. Most participants had been born at home (62.5%; 5/8), while 37.5% (3/8) had been born at the hospital. Most participants 75.0% were multigravida and 25.0% were primigravida. Among women who were HBsAg-positive, 50.0% (4/8) had a history of blood transfusion, 62.5% (5/8) had a history of sharing sharp instruments, all had a history of unprotected sexual intercourse, none had any history of drug abuse by injection, 87.5% (7/8) had a history of hospital admission, 37.5% (3/8) had a history of abortion or miscarriage, 62.5% (5/8) had a history of tooth extraction, 50.0% (4/8) had multiple sexual partners and none had any history of body piercing.

**TABLE 2:** Overall prevalence of HBsAg and HBeAg positivity among participants attending antenatal at Kyazanga Health Centre IV, South Western Uganda, between April 2021 and June 2021.

Variable	Frequency	Proportion (%)	95% Confidence interval
HBsAg	8/384	2.1	1.0–4.1
HBeAg	5/8	62.5	20.0–91.0

**TABLE 3:** Prevalence of HBV infection by risk factor among pregnant women attending Kyazanga Health Centre IV, South Western Uganda, between April 2021 and June 2021.

Risk factors	*N*	Prevalence of HBsAg (%)
**Age category**		
< 24	2	25.0
24–35	6	75.0
36–48	0	0.0
**Place of residence**		
Urban	5	62.5
Rural	3	37.5
**Level of education**		
Primary	5	62.5
Secondary	2	25.0
Tertiary	1	12.5
**Place of birth**		
Others	0	0.0
Home	5	62.5
Hospital	3	37.5
**Gravidity**		
Primagravida	2	25.0
Multigravida	6	75.0
Grand multigravida	0	0.0
**HIV status**		
Positive	0	0.0
Negative	8	100.0
**History of blood transfusion**		
Yes	4	50.0
No	4	50.0
**History of sharing sharp instruments**		
Yes	5	62.5
No	3	37.5
**History of unprotected sexual intercourse**		
Yes	8	100.0
No	0	0.0
**History of injection drug use**		
Yes	0	0.0
No	8	100.0
**History of hospital admission**		
Yes	7	87.5
No	1	12.5
**History of abortion or miscarriage**		
Yes	3	37.5
No	5	62.5
**History of tooth extraction**		
Yes	5	62.5
No	3	37.5
**Multiple sex partners**		
Yes	4	50.0
No	4	50.0
**History of body piercing**		
Yes	0	0.0
No	8	100.0

### Risk factors for hepatitis B virus infection

On univariate analysis, place of birth (odds ratio: 1.06; *p* = 0.018), a history of blood transfusion (odds ratio: 1.037; *p* = 0.043) and a history of hospital admission (odds ratio: 1.03; *p* = 0.04) were associated with HBV infection (Table 4). On multivariate analysis, no variable was significantly associated with HBV infection.

**TABLE 4:** Risk factors predisposing pregnant women to HBV at Kyazanga Health Centre IV, South Western Uganda, between April and June 2021.

Risk factor	Univariate analysis			Multivariate analysis		
	Unadjusted odds ratio	95% confidence interval	*p*-value	Adjusted odds ratio	95% confidence interval	*p-*value
**Age**						
< 24	Ref	-	-	Ref	-	-
24–35	1.020	0.99–1.05	0.228	2.430	0.48–12.25	0.280
36–48	0.986	0.94–1.03	0.530	NA	-	-
**Place of residence**						
Rural	Ref	-	-	Ref	-	-
Urban	1.010	0.98–1.04	0.514	1.610	0.38–6.85	0.516
**Education level**						
Primary	Ref	-	-	Ref	-	-
Secondary	0.997	0.966–1.03	0.837	1.199	0.229–6.27	0.830
Tertiary	1.040	0.97–1.12	0.251	0.310	0.03–2.80	0.295
**Place of birth**						
Other	Ref	-	-	Ref	-	-
Home	1.060	1.01–1.10	0.018	0.670	0.23–1.92	0.460
Hospital	1.010	0.97–1.05	0.535	NA	-	-
**Gravidity**						
Prime	Ref	-	-	Ref	-	-
Multi	1.010	0.98–1.046	0.375	0.506	0.10–2.55	0.409
Grand multi	0.985	0.94–1.04	0.562	NA	-	-
**HIV status**						
Negative	Ref	-	-	Ref	-	-
Positive	0.979	0.885–1.08	0.678	0.302	0.00	0.993
**History of blood transfusion**						
No	Ref	-	-	Ref	-	-
Yes	1.037	1.00–1.07	0.043	3.880	0.95–15.88	0.059
**History of sharing sharp materials**						
No	Ref	-	-	Ref	-	-
Yes	1.000	0.97–1.03	0.963	1.030	0.24–4.39	0.963
**History of unprotected sexual intercourse**						
No	Ref	-	-	Ref	-	-
Yes	1.000	-	-	1.000	-	-
**History of injection drug use**						
No	Ref	-	-	Ref	-	-
Yes	0.978	0.917–1.04	0.504	4.480	0.00	0.995
**History of hospital admission**						
No	Ref	-	-	Ref	-	-
Yes	1.030	1.0–1.06	0.040	6.780	0.83–55.6	0.075
**History of abortion or miscarriage**						
No	Ref	-	-	Ref	-	-
Yes	1.008	0.977–1.04	0.601	1.460	0.34–6.26	0.602
**History of tooth extraction**						
No	Ref	-	-	Ref	-	-
Yes	1.021	0.99–1.05	0.165	2.690	0.63–11.40	0.181
**Multiple sex partners**						
No	Ref	-	-	Ref	-	-
Yes	1.008	0.979–1.038	0.576	1.490	0.37–6.05	0.577
**History of body piercing**						
No	Ref	-	-	Ref	-	-
Yes	0.978	0.91–1.049	0.540	4.520	0.00	0.995

NA, not available; Ref, reference value

## Discussion

### Prevalence of HBV infection

We report the prevalence of HBsAg at 2.1% among pregnant women attending antenatal care at Kyazanga HCIV, Lwengo District, Uganda. The epidemiology of HBV infection can be described in terms of the prevalence of HBsAg positivity in a population. This can be broadly classified as high (> 8.0% HBsAg prevalence), intermediate (2.0% – 7.0% HBsAg prevalence) and low (< 2.0% HBsAg prevalence).^12^ This study reports intermediate endemicity of HBsAg and high prevalence of HBeAg of 62.5% (5/8) among the HBsAg-positive women, which indicates a high risk of perinatal transmission.

In comparison to other studies done within Uganda, the prevalence reported in this study is comparable to infection levels reported at Mbarara Regional Referral Hospital of 2.5% in 2018^13^ and 3.12% in 2019.^6^ The finding of our study is also comparable to 2.9% prevalence which was reported at Mulago Regional Referral Hospital.^5^ This similarity may be because the same risk group was studied and the tests used to ascertain HBV infection were similar (i.e., rapid tests). However, this study’s result does not correlate with the reported prevalence of 11.8% in two hospitals located in Northern Uganda where similar diagnostic methods were used.^7^ This high prevalence of hepatitis B in Northern Uganda could be due to a difference in socio-demographic factors like level of education. A majority of people in Northern Uganda are said to have not gone beyond primary seven.^14^

Within the East African countries, this study closely correlates with the finding of 3.8% in Mbagathi District in Kenya in 2014,^15^ 3.8% at Nyamagana District Hospital in Mwanza, Tanzania in 2014,^16^ 3.9% in Dar es Salaam in 2014^17^ and 3.9% in Rwanda in 2018.^18^ It is lower than the reported prevalence of HBsAg at Khartoum teaching hospital, Sudan, 7.5% in 2010,^19^ and Juba teaching hospital, South Sudan, 11.0% in 2012.^20^ This intra-regional variation could be due to geographical variation, sample size and laboratory methods used for diagnosis.

The prevalence of HBeAg positivity of 62.5% among HBsAg-positive pregnant women reported in this study is higher than the prevalence reported by a study in Northern Uganda at 14.9%.^7^ HBsAg-positive mothers who test positive for HBeAg are known to be highly infectious as the virus is actively replicating.^21^ Therefore, they have a 40.0% to 90.0% risk of transmitting the HBV infection to their babies at birth.^22^ However, our study did not test for anti-HBe or anti-HBc antibody levels, which would further help predict the risk of perinatal transmission in these women.

### Risk factors for HBV infection

None of the risk factors studied had statistically significant associations with HBsAg positivity. This concurs with studies done in Buea in Cameroon in 2012,^23^ and Benin City in Nigeria in 2009.^24^ However, not all of the risk factors considered by those studies were assessed in the current study. We also report here that there was a high prevalence of HBsAg among pregnant women aged 24–35 years, although the association with HBsAg positivity was not stastically significant; this agrees with a study done in Egypt between 2010 and 2011.^25^ This observation of increasing age with HBsAg infection can be attributed to increased likelihood of contracting HBV during each cycle of pregnancy. However, this contrasts with findings from a study done by Bayo and his colleagues in 2012, who reported a high prevalence of HBV infection among pregnant women aged 20 years old or less in Northern Uganda.^7^

The current study also found no association between participants’ history of blood transfusion and HBsAg positivity. This agrees with several studies conducted in various places, including Mbarara Regional Referral Hospital in Uganda in 2018^13^ and 2019,^6^ in Egypt between 2010 and 2011,^25^ in Mulago, Uganda between 2018 and 2019,^5^ and Juba teaching hospital, South Sudan in 2012.^20^ This finding contrasts with studies done in Khartoum, Sudan, in 2010 which found a positive association between participant’s history of blood transfusion and HBsAg positivity.^19^ This observation may be due to implementation of good blood screening strategies for transfusion-transmissible infections in blood bank donations, reducing incidence of infection by blood transfusion.

This study showed a high prevalence of HBsAg positivity among the multigravida pregnant women, although the association was not statistically significant. This contrasts with a study done in Mwanza, Tanzania, in 2014, which showed that multigravidity is associated with HBsAg.^16^ It is thought that chances of exposure to HBV increase as one progresses through the cycle of pregnancy from conception to child birth, and the same happens as the gravidity rises from primagravidity to multigravidity.

History of hospital admission was not found to be significantly associated with HBV infection. This contrasts with a study from Egypt between 2010 and 2011, which found a significant association between hospital admission and HBV infection, since hospital admission may expose pregnant women to surgical procedures which can lead to HBV infection.^25^

Neither a history of abortion nor history of multiple sexual partners were significantly associated with HBsAg positivity in this study. However other studies have reported significant association of hepatitis B infection with history of abortion and multiple sexual partners.^26,27^ This may be due to increases in sexual activity and sexual contact creating more opportunities for acquiring HBV infection This needs further study to test the level of this significance in this area.

No significant association with HBV infection was found with a history of injection drug abuse, body piercing or body tattooing among these women. This is in agreement with a study conducted in southwestern Nigeria in 2013.^28^ However, these factors are considered to increase the chances of becoming infected with HBV.^29^ The lack of a significant association may be attributed to the fact that practices of drug abuse by injection and tattooing are uncommon in this study setting.

### Recommendations

In view of high HBeAg positivity among HBsAg-positive pregnant women, there is need for routine screening of pregnant women for HBsAg and HBeAg to predict the risk of perinatal transmission and strengthen immunisation strategies for babies at risk. There is also a need to adapt treatment and prevention strategies to achieve the world target of free communities without HBV infection.

### Limitations

This study did not explore all the risk factors that may predispose pregnant women to HBV. For example, information about a history of jaundice, vulvular ulcerations, history of sexually transmitted infection, and a family history of HBV were not collected. The self-reported risk factor data used in this study could be subject to recall bias.

### Conclusion

Hepatitis B virus was found to have intermediate endemicity among the pregnant women attending antenatal care at Kyazanga HCIV. However, this is not too low to cause morbidity and mortality among the population. None of the proposed risk factors were significantly associated with HBV infection; explanations for this observation need to be explored in future.
